# P-1013. Incidence and Risk Factors for Fungal Infection after CD19-targeted Chimeric Antigen Receptor T cell Therapy for Non-Hodgkin Lymphoma

**DOI:** 10.1093/ofid/ofae631.1203

**Published:** 2025-01-29

**Authors:** Charles Gaulin, Roy F Chemaly, Ying Jiang, Jeremy Ramdial, Sairah Ahmed, Gabriella Rondon, Ella Ariza Heredia, Amy Spallone, Swaminathan Iyer, Partow Kebriaei, Samer Srour, Elizabeth Shpall, Loretta Nastoupil, Fareed Khawaja

**Affiliations:** The University of Texas MD Anderson Cancer Center, Houston, Texas; University of Texas MD Anderson Cancer Center, Houston, TX; The University of Texas MD Anderson Cancer Center, Houston, Texas; University of Texas MD Anderson Cancer Center, Houston, TX; The University of Texas MD Anderson Cancer Center, Houston, Texas; The University of Texas MD Anderson Cancer Center, Houston, Texas; The University of Texas MD Anderson Cancer Center, Houston, Texas; University of Texas MD Anderson Cancer Center, Houston, TX; The University of Texas MD Anderson Cancer Center, Houston, Texas; MD Anderson Cancer Center, Houston, Texas; The University of Texas MD Anderson Cancer Center, Houston, Texas; The University of Texas MD Anderson Cancer Center, Houston, Texas; The University of Texas MD Anderson Cancer Center, Houston, Texas; The University of Texas MD Anderson Cancer Center, Houston, Texas

## Abstract

**Background:**

Chimeric antigen receptor T cell therapy (CAR T) is highly effective for the treatment of non-Hodgkin lymphoma (NHL); however, recipients are at risk of infectious complications. Infections following CAR T are predominantly bacterial or viral; nevertheless, a subset of recipients develop invasive fungal disease (IFD). The limited evidence guiding antifungal prophylaxis (PPx) led us to investigate the incidence and risk factors for IFD at our center.

**Figure:**
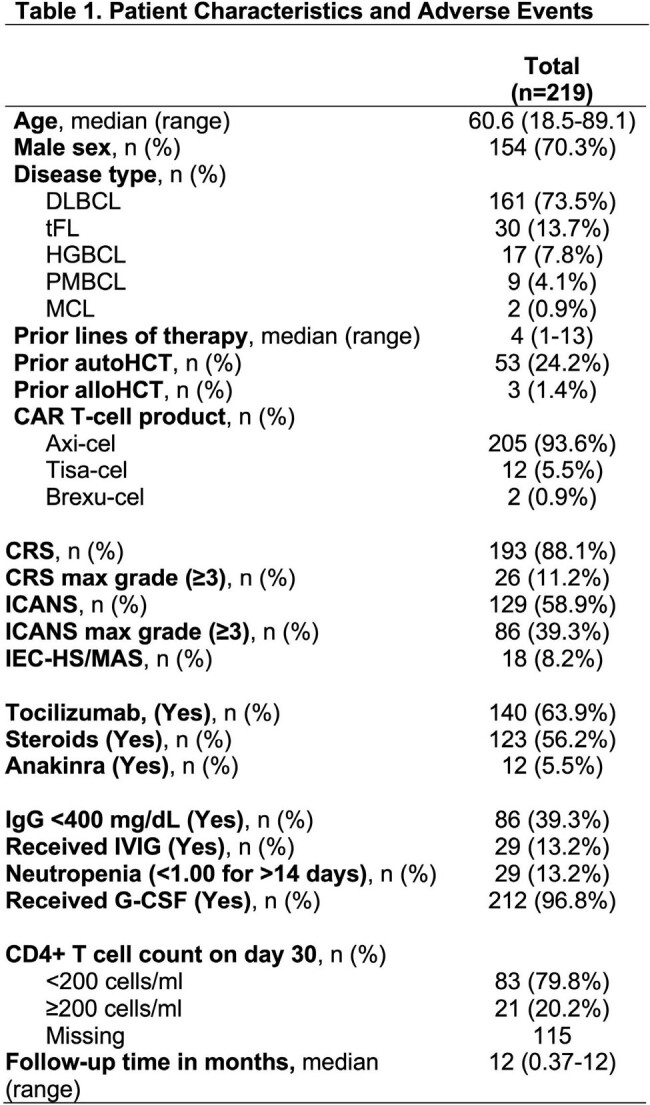

Legend: DLBCL=diffuse large B-cell lymphoma; MCL=mantle cell lymphoma; tFL=transformed follicular lymphoma; PMBCL=primary mediastinal large B-cell lymphoma; HGBCL= high-grade B-cell lymphoma; autoHCT=autologous hematopoietic cell transplant; alloHCT=allogeneic hematopoietic cell transplant; CAR =chimeric antigen receptor; Axi-cel=axicabtagene ciloleucel; Brexu-cel=brexucabtagene autoleucel; Tisa-cel=tisagenlecleucel; CRS=cytokine release syndrome; ICANS= immune effector cell-associated neurotoxicity syndrome; IEC-HS/MAS= immune effector cell-associated HLH-like syndrome/macrophage activation syndrome; IgG=immunoglobulin G; G-CSF=granulocyte colony-stimulating factor

**Methods:**

Adults with NHL who received commercial CAR T between January 2018 and February 2021 at MD Anderson Cancer Center were retrospectively identified. Demographics, oncologic history, and complications related to therapy were collected. Consensus definitions were used to define probable and proven IFD. Patients were observed for 1 year after CAR T or until death, whichever occurred first. Routine antifungal PPx was administered per institutional protocol. This database was built with funds from Merck.

**Figure:**
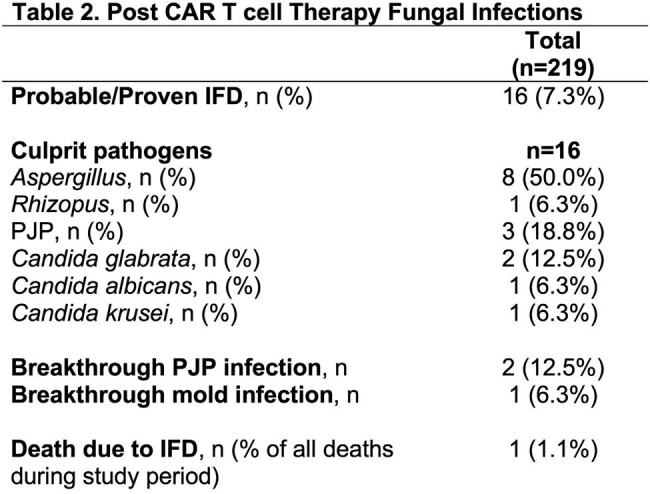

Legend: IFD=invasive fungal disease; PJP=Pneumocystis jirovecii pneumonia

**Results:**

A total of 219 patients were identified (Table 1). Most patients had an underlying diagnosis of diffuse large B cell lymphoma (73.5%) and received axicabtagene ciloleucel (93.6%). Therapy related toxicities included: cytokine release syndrome (CRS) (88.1%), immune effector cell-associated neurotoxicity syndrome (ICANS) (58.9%) and immune effector cell-associated hemophagocytic lymphohistiocytosis-like syndrome (IEC-HS) (8.2%). Sixteen patients (7.3%) developed IFD, including 9 (4.1%) mold infections (Table 2). In univariate analysis (Table 3a), IEC-HS, grade 4 CRS, grade 4 ICANS, and any infection within 30 days prior to CAR T were associated with IFD. In multivariate analysis (Table 3b), grade 4 CRS, grade 4 ICANS, and any infection within 30 days prior to CAR T were independent predictors for IFD. Factors associated with mold infections in univariate analysis (Table 3c) included IEC-HS, grade 4 CRS, grade 4 ICANS, steroid use, prior hematopoietic cell transplantation (HCT), and any infection documented within 30 days prior to CAR T.

**Figure:**
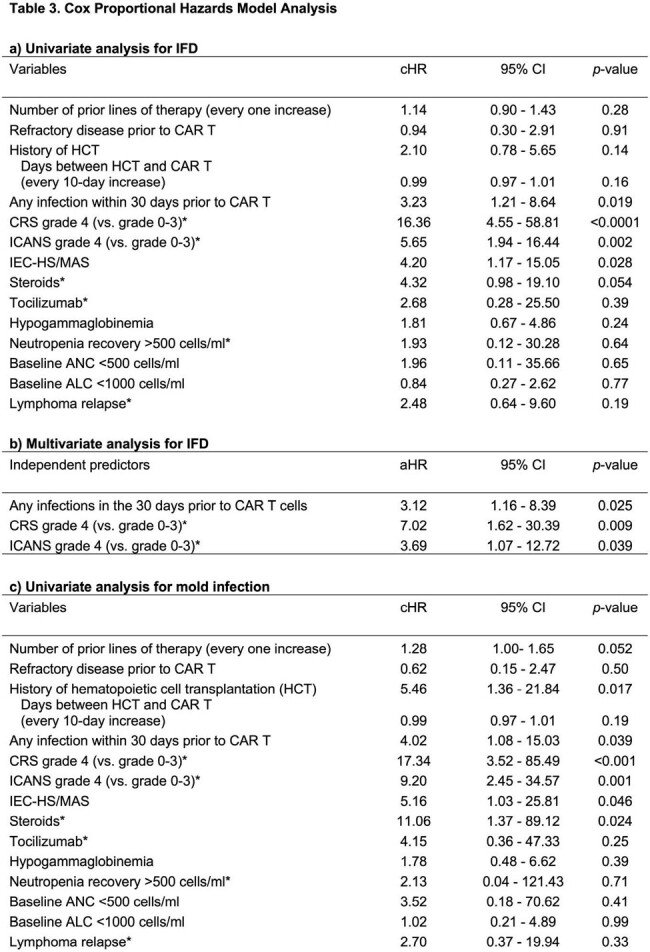

Legend: IFD=invasive fungal disease; cHR= Crude hazard ratio; 95% CI= 95% Confidence interval; aHR= Adjusted hazard ratio; CAR T=Chimeric antigen receptor T cell therapy; HCT=autologous hematopoietic cell transplant; CRS=cytokine release syndrome; ICANS= immune effector cell-associated neurotoxicity syndrome; IEC-HS/MAS= immune effector cell-associated HLH-like syndrome/macrophage activation syndrome; ANC=absolute neutrophil count; ALC=absolute lymphocyte count. Note:* indicates the variable was treated as a time-dependent variable in the analysis. Multivariate analysis on mold infection was not performed due to the small number of the events.

**Conclusion:**

IFD after CAR T for NHL is uncommon. Higher grade toxicities and their treatment, as well as prior HCT and prior infections, may identify those at highest risk. Additional studies are needed to individualize antifungal PPx strategies in this population.

**Disclosures:**

**Charles Gaulin, MBBS**, ADC Therapeutics: Honoraria|DeciBio: Advisor/Consultant|Sanofi: Honoraria **Roy F. Chemaly, MD/MPH**, AiCuris: Advisor/Consultant|AiCuris: Grant/Research Support|Ansun Pharmaceuticals: Advisor/Consultant|Ansun Pharmaceuticals: Grant/Research Support|Astellas: Advisor/Consultant|Eurofins-Viracor: Grant/Research Support|InflaRX: Advisor/Consultant|Janssen: Advisor/Consultant|Karius: Advisor/Consultant|Karius: Grant/Research Support|Merck/MSD: Advisor/Consultant|Merck/MSD: Grant/Research Support|Moderna: Advisor/Consultant|Oxford Immunotec: Advisor/Consultant|Oxford Immunotec: Grant/Research Support|Roche/Genentech: Advisor/Consultant|Roche/Genentech: Grant/Research Support|Shinogi: Advisor/Consultant|Takeda: Advisor/Consultant|Takeda: Grant/Research Support|Tether: Advisor/Consultant **Fareed Khawaja, MBBS**, Eurofins Viracor: Grant/Research Support|Symbio: Grant/Research Support

